# The effect of combined cognitive behavioral therapy and rehabilitation training on negative emotions of patients with vestibular dysfunction: a real-world cohort study

**DOI:** 10.3389/fpubh.2026.1923062

**Published:** 2026-09-09

**Authors:** Lu Gao, Meifang Huang, Xiaoxue Yang, Xiaojing Liang, Ling Li, Liqiong Yu, Shaoqin Ma, Heng Xiao

**Affiliations:** 1 Department of Otolaryngology Head and Neck Surgery, the First Affiliated Hospital, Fujian Medical University, Fuzhou, China; 2 Department of Otolaryngology Head and Neck Surgery, National Regional Medical Center, Binhai Campus of the First Affiliated Hospital, Fujian Medical University, Fuzhou, China; 3 Fujian Institute of Otorhinolaryngology, The First Affiliated Hospital, Fujian Medical University, Fuzhou, China; 4 The Sixth Medical Center of PLA General Hospital, National Clinical Research Center for Otolaryngologic Diseases, Beijing, China

**Keywords:** cognitive behavioral therapy, negative emotions, real-world study, rehabilitation training, vestibular dysfunction

## Abstract

**Purpose:**

This study evaluated the association of combined cognitive behavioral therapy (CBT) and rehabilitation training with improvements in vestibular dysfunction and negative emotions based on real-world clinical practice.

**Methods:**

This single-center, real-world, retrospective cohort study recruited 100 patients with vestibular dysfunction and negative emotions from a tertiary hospital in China between August 2020 and October 2024. Patients were divided into two groups based on actual treatment received: the conventional care group (*n* = 50) received standard medication and health education, while the combined intervention group (*n* = 50) additionally underwent a 3-month program of CBT combined with rehabilitation training. Primary outcomes included Visual Analog Scale (VAS), Dizziness Handicap Inventory (DHI), and Somatic Symptom Scale (SSS) scores at 3 months. Secondary outcomes included the above scales at 1 month, along with Generalized Anxiety Disorder Scale-7 (GAD-7) and Patient Health Questionnaire-9 (PHQ-9) scores.

**Results:**

At 3 months, the combined intervention group showed significantly greater improvement in VAS, DHI, and SSS scores compared to the conventional care group (all *p* < 0.05). At 1 month, the combined group demonstrated early superiority in the emotional and functional dimensions of the DHI. GAD-7 and PHQ-9 scores showed significantly greater psychological improvement in the combined group at both 1 and 3 months (*p* < 0.01).

**Conclusion:**

In real-world settings, CBT combined with rehabilitation training was associated with greater improvements in dizziness, somatic symptoms, and anxiety/depression in vestibular dysfunction patients with negative emotions compared with conventional care. Most outcomes improved early, whereas the physical component of dizziness-related disability showed a later between-group separation, which supports the potential value of integrated psychosomatic care models.

## 1 Introduction

Vestibular dysfunction arises from damage to the peripheral or central vestibular system and is primarily characterized by dizziness, vertigo, vestibulo-ocular dysfunction and postural imbalance (1). These symptoms typically persist for periods ranging from several days to several years and may occur individually or in combination; they not only severely impact daily activities, mental health and overall quality of life, but also increase the risk of falls and fall-related injuries (2–4). Approximately 30–40% of the general population have experienced dizziness, with a prevalence exceeding 35% among adults aged 40 and over and an annual incidence of 11.9% (5, 6). The associated socio-economic burden is significant, including increased utilisation of healthcare resources, prolonged sick leave and reduced labour productivity (7). In addition to physiological effects, there is a complex interplay between vestibular dysfunction and negative emotional states. Research indicates that vestibular dysfunction frequently co-occurs with anxiety and depression, with a possible bidirectional association and a higher comorbidity rate than in the general population (8, 9). The underlying mechanisms may involve vestibulo-limbic pathway interactions, through which abnormal vestibular signals modulate functional activity in limbic structures including the amygdala and hippocampus, thereby contributing to emotional dysregulation (10). Furthermore, persistent vertigo and postural instability often trigger kinesiophobia and a catastrophic interpretation of bodily sensations, leading to functional avoidance, whereby patients gradually reduce their physical activity and social participation (11). This reduction in vestibular stimulation delays the central compensatory process, thereby exacerbating symptoms of anxiety and depression and creating a self-perpetuating vicious cycle of ‘vestibular symptoms-emotional distress-functional avoidance-delayed compensation’ (8, 12).

Vestibular rehabilitation therapy (VRT) (3) remains one of the key treatments for vestibular dysfunction. Based on the principles of neuroplasticity, VRT improves vestibular symptoms, enhances postural stability and promotes central compensation through gaze stabilisation, habituation and balance training. However, VRT has notable limitations (13–15), including incomplete symptom relief in some patients, significant inter-individual variability in treatment response, poor adherence to training programmes, and psychological factors such as anxiety, depression and catastrophic thinking, which significantly influence rehabilitation outcomes. Moreover, traditional VRT protocols predominantly address physical symptoms while insufficiently attending to patients co-occurring negative emotions, which may explain why symptom relief is often incomplete and why adherence to rehabilitation training is frequently compromised. These limitations suggest that relying solely on physiological compensation often fails to achieve optimal recovery outcomes. Cognitive Behavioral Therapy (CBT) offers a complementary intervention by addressing the cognitive and behavioral factors that maintain dizziness-related disability (16). In patients with vestibular dysfunction, CBT can specifically alleviate dizziness-related fears, symptom catastrophising, hypervigilance to somatic cues, and avoidance behaviours that hinder vestibular compensation, thereby breaking the aforementioned vicious cycle (17). The integration of CBT with VRT is conceptually synergistic: while VRT primarily drives neurophysiological compensation through exercise therapy, CBT targets the maladaptive cognitive-behavioral responses that hinder engagement with rehabilitation and undermine functional recovery (18).

The combination of the two may produce additive or synergistic effects on vestibular symptoms and psychological outcomes (19, 20). Several studies have investigated the efficacy of combined CBT and VRT treatment (21–23), with preliminary evidence suggesting improvements in the severity of dizziness, anxiety and functional impairment. However, the existing literature has numerous limitations, including small sample sizes, study populations predominantly drawn from highly selected, single-centre cohorts, short follow-up periods, and considerable heterogeneity in intervention protocols (16, 24, 25). More crucially, most of the existing evidence originates from strictly controlled, idealised research settings, whilst implementation studies on efficacy in routine clinical practice are extremely scarce. In a routine clinical setting, nurses are the clinical staff who have the most continuous and close contact with patients. Existing evidence (26–29) indicates that nurse-led CBT and nurse-delivered psychological interventions demonstrate good feasibility, acceptability and clinical efficacy in the fields of chronic disease management and rehabilitation, further supporting the feasibility of a nurse-led integrated CB-VR model in real-world clinical practice.

The present study was therefore designed to address this evidence gap. Specifically, we implemented a nurse-delivered integrated CB-VR model and evaluated its real-world effectiveness in improving negative emotions (anxiety and depression) and functional disability among patients with vestibular dysfunction accompanied by emotional distress, using data collected within a routine clinical care framework. The specific study objective was to compare the effects of the nurse-delivered CB-VR model versus routine care on: (i) vestibular symptom severity (VAS, DHI, SSS); (ii) anxiety (GAD-7); and (iii) depression (PHQ-9) at 1-month and 3-month follow-up, while controlling for baseline demographic and clinical confounders in a real-world cohort setting. We anticipate that the findings may inform future clinical practice and policy development regarding mind–body integrated nursing services, although we acknowledge that confirmatory research remains warranted.

## 2 Methods

### 2.1 Research design

This study was conducted as a single-centre, real-world, retrospective cohort study. The research question was to evaluate the effectiveness of combined cognitive behavioral therapy and rehabilitation training in improving vestibular dysfunction and negative emotions in real-world clinical practice. To enhance the quality and transparency of health research, the study was reported in accordance with the Strengthening the Reporting of Observational Studies in Epidemiology (STROBE) checklist for cohort studies. The study was conducted in accordance with the Declaration of Helsinki and was approved by the ethics committee (approval numbers: [2013]62 for original data collection; [2023]388 for this analysis). Written informed consent was obtained from all participants at the time of original data collection, and the ethics committee waived the requirement for re-consent for this retrospective analysis. Patient data were anonymised prior to analysis. All outcome assessments were part of routine clinical care and were extracted retrospectively for this analysis.

### 2.2 Research subjects and locations

By querying the electronic medical record system, clinical data were consecutively enrolled from 371 patients with vestibular dysfunction who visited the Department of Otolaryngology-Head and Neck Surgery at a Grade A tertiary general hospital in China between August 2020 and October 2024. Inclusion criteria were: (1) clinically diagnosed vestibular dysfunction, including PPPD (Bárány Society criteria, 2017) (30), Ménière’s disease (AAO-HNS criteria, 2015) (31), and vestibular migraine (ICHD-3 criteria, 2012) (32); (2) Generalized Anxiety Disorder (GAD-7) score ≥5 and ≤18; (3) Patient Health Questionnaire (PHQ-9) depression screening score ≥5 and ≤19; (4) Age ≥18 years and <80 years. Exclusion criteria were: (1) Presence of structural central nervous system lesions (e.g., tumors, cerebrovascular lesions, or demyelinating diseases), inner ear malignancies, or cerebellopontine angle tumors (e.g., acoustic neuroma) that could confound diagnosis or treatment response; (2) Patients with severe cervical spine lesions or fractures; (3) Individuals undergoing intensive anti-anxiety or antidepressant therapy; (4) Those with severe psychiatric disorders or cognitive impairment-related conditions; (5) Incomplete clinical documentation at baseline. Through electronic medical record review and repeated questionnaire assessments (baseline, 1 month, and 3 months), it was confirmed that no patients in either group used psychotropic medications (SSRIs, SNRIs, benzodiazepines) or vestibular suppressants during the entire study period. A total of 100 patients meeting the aforementioned criteria were ultimately enrolled. They were naturally grouped based on the treatment regimens they actually received in real clinical settings: patients receiving only medication and health education were assigned to the conventional care group, while those receiving cognitive behavioral therapy combined with rehabilitation training in addition to conventional care were assigned to the combined intervention group. To further clarify the basis of this natural grouping, we reviewed the clinical records of all patients who, based on standardized baseline assessments, met the eligibility criteria for the CB-VR intervention and had no contraindications. Since CB-VR was not randomly assigned, patients were grouped according to whether they actually initiated the intervention after baseline evaluation. Patients who initiated CB-VR were assigned to the CB-VR group; those who did not—despite meeting eligibility criteria—were assigned to the usual care group. Reasons for non-initiation documented in the clinical records included patient or family refusal, geographic distance or transportation difficulties, clinician non-referral, and limited treatment resources, scheduling conflicts, and other reasons. All patients underwent baseline assessments at their initial visit and completed follow-up assessments at 1 month and 3 months.

### 2.3 Research methods

Conventional Care Group: Received standard drug therapy and health education interventions. Patients in the conventional care group received standard pharmacotherapy for vestibular dysfunction, specifically betahistine mesylate tablets, 12 mg three times daily, for 12 weeks, combined with standardized health education. This regimen represents the standard pharmacotherapy for vestibular disorders in our institution. In addition, patients received standardized health education covering disease understanding, family caregiver support, and safety measures during vertigo episodes to prevent falls.

Combined Intervention Group: In addition to the standard care, this group received CBT combined with personalized vestibular rehabilitation training, delivered by a multidisciplinary team comprising two clinical physicians specializing in dizziness from the Department of Otolaryngology-Head and Neck Surgery, two specialized nurses, and one vestibular function technician. The physicians were responsible for diagnosis, patient referral, and medical oversight; the technician performed baseline vestibular function tests; and the specialist nurses delivered the CBT and rehabilitation components through a nurse-led vestibular rehabilitation clinic. Building upon personalized VRT, trained specialist nurses integrated core CBT components, including a three-phase structured cognitive behavioral therapy program and individualized vestibular rehabilitation training courses in Table 1.

**Table 1:** CBT combined with personalized vestibular rehabilitation intervention protocol.

Time	Theme	Specific details
Stage 1 (1–4 weeks)	CBT: Psychological education + cognitive restructuring	① Establish a good therapeutic relationship through symptom-oriented treatment. ② Help patients and their families understand disease-related knowledge: the pathogenesis of vertigo, mechanisms of balance maintenance, disease progression, and prognosis-related information, etc. ③ Psychophysiological knowledge of vestibular dysfunction, cognitive understanding of dysfunction, and psychological education on avoidance behaviors;understanding the inevitable connection between “dizziness-emotion-cognition.” ④ Learn to identify situations that trigger dizziness (e.g., crosswalks, supermarkets, etc.) and catastrophic thinking (e.g., dizziness = “I’m going to fall,” going out = “I’m going to get dizzy,” etc.). ⑤ Simulated behavior experiments and verification of fear predictions (e.g., “Will turning my head to the right/left definitely make me dizzy?”). ⑥ Cognitive restructuring: using evidence to refute catastrophic thinking and fear-based behavior predictions. ⑦ Lifestyle and healthy sleep guidance: Develop good habits and healthy sleep patterns.
	Personalized vestibular rehabilitation training	① Dynamic and static gaze stability training. ② Graded gait balance training.
	Homework	① Keep a diary of dizziness (time, situation, intensity). ② Record automatic thoughts in a three-column table (event, automatic thought, emotion, and behavioral response). ③ Take a 20–30 min walk every day accompanied by family members.
Stage 2 (5–8 weeks)	CBT: Graded exposure + behavioral intervention	① Assess patients’ symptoms of vestibular dysfunction, balance function, and mood, and adjust intervention goals and plans in a timely manner based on the results. ② Identify the context of the cycle of dizziness, irrational cognitions, and personal behavioral patterns (such as avoidance behavior, fear of falling, social barriers, etc.) in patients based on dizziness diaries and automatic thought three-column tables. ③ Help patients and their families understand the cycle of dizziness: mainly caused by high-risk postures and maladaptive behaviors. Guide patients to develop a positive understanding and thinking about the disease and correct cognitive biases. ④ Based on the vicious cycle of dizziness, create a fear scale and classify exposure from low risk to high risk (e.g., going out → crowded shopping mall). ⑤ Establish a graded exposure desensitization strategy, conduct desensitization exercises for dizziness symptoms and visual stimuli, as well as daily functional activities and alternative training, and provide guidance on coping techniques for exacerbation of dizziness. ⑥ Guide them through muscle relaxation exercises, mindful breathing, listening to classical, convey positive rehabilitation beliefs, and reinforce the patient’s confidence.
	Upgraded personalized vestibular rehabilitation training	① Complex dynamic balance (such as turning the head while walking). ② Add visual reinforcement actions or dual-task training that combines cognitive tasks (add calculation tasks during training). ③ Functional exercises related to dizziness diaries (such as jogging, badminton, etc.).
	Homework	① Keep a diary of dizziness (time, situation, intensity). ② Record automatic thoughts in a three-column table (event, automatic thought, emotion, and behavioral response). ③ Graded exposure therapy exercises and desensitization training.
Stage 3 (9–12 weeks)	CBT: Lifestyle reconstruction + relapse prevention	① Conduct a summary of the treatment phase: identify remaining issues, teach patients and their families how to recognize warning signs of vestibular dysfunction, develop a list of response strategies, and build long-term coping skills to prevent recurrence. ② Self-efficacy enhancement: Encourage patients to apply cognitive changes in their feelings, cognition, and behavior to their daily lives and engage in self-guided training. ③ Emphasize the importance of resuming normal life starting now.
	Advanced personalized vestibular rehabilitation	① High-intensity comprehensive training. ② Simulate real-life scenarios (such as bus jolts and spinning in place).
	Homework	① Gradually transition from dependence on medical intervention and guidance to self-management. ② Independently develop weekly rehabilitation training and exercise plans.

#### 2.3.1 Personalized vestibular rehabilitation training methods

Patients in the combined intervention group received rehabilitation training demonstrations, guidance, and personalized rehabilitation plans developed by vestibular rehabilitation nurses based on baseline assessments at the initial visit, in addition to routine medication and health education interventions (18, 33). This included multimodal training such as gaze stability exercises, graded gait balance training, habituation training, and functional exercises. The degree of target movement completion, exercise frequency, movement speed and amplitude, and duration were all personalized and adjusted based on symptom progression and training duration. After mastering the fundamental movement techniques, patients were informed of the importance of fall prevention and protective measures. They were instructed to adhere to daily rehabilitation training for no fewer than two sessions, with a total daily practice duration of no less than 12 min. The entire treatment cycle spans 3 months.

#### 2.3.2 Cognitive behavioral intervention therapy

Building upon personalized vestibular rehabilitation training, specialized nurses who have undergone dedicated training integrate core CBT components to develop structured psychosomatic intervention protocols. Specifically including: (1) Through psychological education (34, 35), explain the cyclical mechanism linking “vestibular symptoms-emotions-cognition” to patients and their families. This helps patients develop accurate disease awareness and employs cognitive restructuring techniques to guide them in identifying and correcting catastrophic thinking. (2) Develop individualized graded exposure plans to gradually reduce avoidance behaviors stemming from vestibular symptoms. (3) Emphasize the importance of “rebuilding life starting now” rather than waiting for symptoms to disappear, teaching acute symptom self-management and long-term coping skills.

To ensure standardization and consistency in intervention delivery, all nurses who delivered the intervention completed a six-month structured training program designed by the department’s dizziness specialist physicians, in consultation with clinical psychologists with CBT expertise. The training covered CBT principles and their application in vestibular rehabilitation, the three-phase protocol (Table 1), assessment and treatment planning, and patient communication skills, using a blended format of clinical shadowing, case discussions, simulations, and supervised practice. Nurses received ongoing supervision from the specialist physicians during training and were required to pass a competency assessment before independently delivering the intervention; regular case discussion meetings were held throughout the study period to maintain consistency. The intervention comprised three structured sessions (baseline, 1 month, and 3 months), each lasting approximately 60–90 min, totaling 3 to 4.5 h of professional contact per patient. All 50 patients in the intervention group attended all three sessions and self-reported completion of daily home exercises.

### 2.4 Assessment tools

The Visual Analogue Scale (VAS) (36) is used to rapidly assess patients’ subjective severity of dizziness during the current episode. This scale ranges from 0 to 10, where 0 indicates no dizziness and 10 represents the most severe level of dizziness. Higher scores indicate greater severity of dizziness, impaired self-care ability, or reduced quality of life. The Dizziness Handicap Inventory (DHI) (37) was further employed to evaluate patients’ dizziness severity, balance dysfunction, and treatment efficacy. Four indices can be calculated: total score (DHI-T), physical symptoms score (DHI-P), emotional symptoms score (DHI-E), and functional limitations score (DHI-F). The DHI total score ranges from 0 to 100 points, with higher scores indicating more severe dizziness or balance impairment. The Somatic Symptom Scale (SSS) (38) was used to assess the severity of somatization in patients with vertigo. Higher scores indicate greater severity of somatic impairment, leading to increased difficulties in work, study, family relationships, and interpersonal interactions. The Generalized Anxiety Disorder-7 (GAD-7) (39) and Patient Health Questionnaire-9 (PHQ-9) (40) were used to assess anxiety and depressive symptoms. Higher scores indicate greater severity of anxiety and depressive mood.

### 2.5 Outcome Indicator

The primary outcome measures were the total scores on the Dizziness Handicap Inventory (DHI), Visual Analogue Scale (VAS), and Somatic Symptom Scale (SSS) at 3 months. Secondary outcomes included scores on primary outcome measures (DHI, VAS, SSS) at 1 month, as well as scores on the GAD-7 and PHQ-9 depression screening scales at 1 and 3 months.

### 2.6 Data collection

All data for this study were collected through retrospective queries of the electronic medical record system at a tertiary-level Class A general hospital in China. To ensure data accuracy and completeness, two researchers trained uniformly conducted independent data extraction and entry using standardized data extraction forms. Data extraction was performed by research staff who were not involved in the intervention delivery. Discrepancies were resolved through discussion or adjudication by a third researcher. All collected quantitative data were entered into pre-designed Excel spreadsheets and double-checked for accuracy.

### 2.7 Statistical methods

Data were analyzed using SPSS (Version 25.0). All analyses were conducted on a per-protocol basis, including only participants who completed the full 3-month follow-up. Continuous variables were tested for normality using the Shapiro–Wilk test. Normally distributed data were presented as mean (standard deviation, SD) and compared between groups using independent samples t-tests. Non-normally distributed continuous and ordinal data were presented as median (interquartile range, IQR) and compared using the Mann–Whitney U test. Categorical variables were analyzed using the chi-square test. A two-way repeated measures analysis of variance (ANOVA) was conducted to evaluate the effects of group (intervention vs. control) and time (baseline, 1 month, and 3 months) on the outcome measures. Mauchly’s test of sphericity was performed, and if violated (*p* < 0.05), the Greenhouse–Geisser correction was applied. The group-by-time interaction was examined. Pairwise comparisons were performed with Bonferroni correction for multiple comparisons. Partial eta squared (η^2^) was reported as a measure of effect size for the ANOVA results. To control for potential baseline imbalances, analysis of covariance (ANCOVA) was performed for the primary outcomes (DHI and SSS total scores at 3 months), with age, educational level, disease duration at first consultation (in months), and the respective baseline score of each outcome as covariates. The same covariate set was used consistently across all ANCOVA models. For VAS, ANCOVA was not performed because the distribution at 3 months was severely skewed due to a strong floor effect; therefore, repeated-measures ANOVA results are reported as the primary analysis for this outcome. Effect sizes for ANCOVA were also reported as partial eta squared (η^2^). All statistical tests were two-tailed, and *p* < 0.05 was considered statistically significant. Given the exploratory nature of this real-world study, the analyses of secondary outcomes and DHI subscales should be considered hypothesis-generating. No adjustment for multiple testing across multiple outcomes or subscales was performed. Results should be interpreted with caution, and confirmatory studies are needed to validate these findings. As this was a retrospective analysis of routinely collected clinical data, prospective trial registration was not obtained.

## 3 Results

### 3.1 Patient flow and baseline characteristics

**Figure 1:**
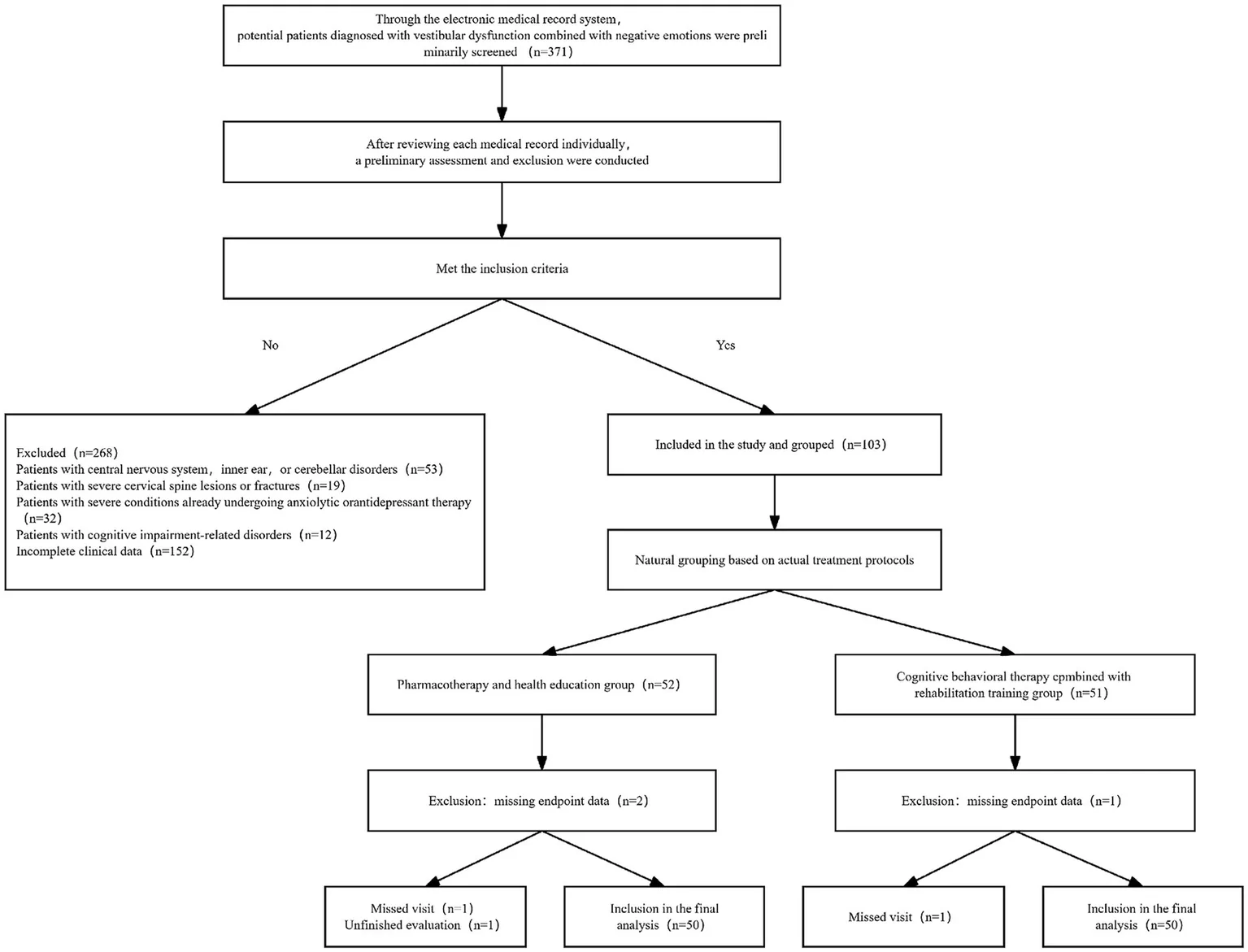
Patient screening, grouping, and dropout flowchart.

The complete process of patient selection, grouping, and dropout in this study is shown in Figure 1. A total of 371 patients with vestibular dysfunction and negative emotions were initially screened, of whom 268 were excluded for not meeting the eligibility criteria (see Figure 1 for detailed exclusion categories). The remaining 103 eligible patients were allocated to the conventional care group (*n* = 52) or the combined intervention group (*n* = 51) based on the treatment regimen they received in routine clinical practice. During the 12-week follow-up period, one patient in the combined intervention group was excluded due to missing endpoint data; two patients in the conventional care group were excluded due to missing endpoint data. The overall attrition rate was 2.9% (3/103), with 2.0% (1/51) in the combined intervention group and 3.8% (2/52) in the conventional care group. Consequently, 100 patients (50 per group) with complete data were included in the final per-protocol analysis. No imputation was performed for missing data.

The baseline demographic and clinical characteristics of the two groups are summarised in Table 2. Formal comparison of baseline characteristics between completers and non-completers was not performed due to the small number of non-completers (*n* = 3). No significant differences were observed between the two groups in terms of age, educational level, duration of illness, diagnostic subtype (*χ*^2^ = 0.728, *p* = 0.695), vestibular function tests (Unterberg and Romberg tests), VAS, DHI total score, DHI subscales (Physical, Emotional, Functional), SSS, GAD-7, or PHQ-9 scores (all *p* > 0.05), indicating that the two groups were well-balanced at baseline. Although no statistically significant baseline differences were observed, the usual care group had marginally higher mean DHI and SSS scores than the combined intervention group. To further control for potential confounding factors and improve statistical efficiency, we included age, educational level, disease duration, and the respective baseline score of each outcome as covariates in the ANCOVA analyses.

**Table 2:** Baseline characteristic variables of the study subjects (*n* = 100).

Project	Conventional care group (*n* = 50)	Combined intervention group (*n* = 50)	*X*^2^/Z/t	*P*
Age(years)	53.38 ± 15.15	51.8 ± 12.89	−0.738	0.460
<50	16	17		
≥50	34	33		
Gender			0.174	0.677
Male	17	19		
Female	33	31		
Duration of illness at first visit (years)	31.05 ± 54.56	24.02 ± 24.96	−0.513	0.608
<0.5	11	17		
0.5–1	18	10		
>1–5	16	22		
>5	5	1		
Diagnosis subtype [*n* (%)]			0.728	0.695
PPPD	21	17		
Menière’s disease	15	18		
Vestibular migraine	14	15		
Level of education			0.225	0.893
Junior high school and below	29	27		
High school (vocational high school)	9	9		
College degree or above	12	14		
Unterberger test			0.102	0.749
Normal	5	6		
Abnormal	45	44		
Romberg test			2.041	0.495
Negative	0	2		
Positive	50	48		
Clinical outcome measures				
VAS	4.02 ± 1.04	3.70 ± 1.07	1.514	0.133
DHI	52.24 ± 12.9	51.82 ± 12.69	0.164	0.87
DHI–Physical	13.56 ± 4.89	13.74 ± 3.42	−0.213	0.831
DHI–Emotional	16.48 ± 5.71	16.60 ± 6.02	−0.139	0.890
DHI–Functional	22.20 ± 6.65	21.48 ± 7.7	0.501	0.618
SSS	44.22 ± 6.58	42.36 ± 7.45	1.323	0.189
GAD-7	6.02 ± 4.24	6.82 ± 4.44	−0.979	0.328
PHQ-9	7.06 ± 4.61	8.28 ± 5.57	−0.958	0.338

### 3.2 Primary outcome measure

The primary outcomes of this study were the total scores on the VAS, DHI-T, and SSS scales at 3 months for both patient groups. Although no statistically significant baseline differences were observed between the two groups, the conventional care group had marginally higher mean DHI and SSS scores than the combined intervention group. To further control for potential confounding and improve statistical efficiency, ANCOVA was performed with age, educational level, disease duration, and the respective baseline score of each outcome as covariates. For the DHI, after adjustment, the estimated marginal mean at 3 months was 29.603 (95% CI: 28.028–31.179) in the conventional care group and 13.857 (95% CI, 12.281–15.432) in the combined intervention group, with a mean difference of 15.747 (95% CI: 13.513–17.981, *F*(1,94) = 195.867, *p* < 0.001, partial η^2^ = 0.676). For the SSS, the adjusted mean was 36.762 (95% CI, 35.363–38.162) in the conventional care group and 24.898 (95% CI, 23.497–26.297) in the combined intervention group, with a mean difference of 11.865 (95% CI: 9.873–13.857, *F*(1,94) = 139.852, *p* < 0.001, partial η^2^ = 0.598).

Repeated-measures ANOVA results. To evaluate the trajectory of changes across baseline, 1 month, and 3 months, two-way repeated-measures ANOVA with Greenhouse–Geisser correction was applied. For VAS, a significant time × group interaction was observed [*F*(1.921, 188.223) = *F*(1.968, 192.826) = 68.854, *p* < 0.001, partial η^2^ = 0.413], indicating that the combined intervention group showed a significantly greater reduction in vertigo symptoms over time compared with the conventional care group. At 3 months, the between—group mean difference for VAS was 2.38(95% CI: 2.079–2.681). The main effects of time [*F*(1.968, 192.826) = 397.228, *p* < 0.001, partial η^2^ = 0.802] and group [*F*(1,98) = 53.408, *p* < 0.001, partial η^2^ = 0.353] were also significant. For DHI, the time × group interaction was significant [*F*(1.414, 138.549) = 67.270, *p* < 0.001, partial η^2^ = 0.407], as were the main effects of time [*F*(1.414, 138.549) = 1013.236, *p* < 0.001, partial η^2^ = 0.912] and group [*F*(1, 98) = 14.805, *p* < 0.001, partial η^2^ = 0.131]. For SSS, a significant time × group interaction was also observed [*F*(1.772, 173.629) = 52.367, *p* < 0.001, partial η^2^ = 0.348], along with significant main effects of time [*F*(1.772, 173.629) = 274.319, *p* < 0.001, partial η^2^ = 0.737] and group [*F*(1, 98) = 37.913, *p* < 0.001, partial η^2^ = 0.279]. All pairwise comparisons were Bonferroni-corrected. Taken together, these results consistently demonstrate that the combined intervention group experienced significantly greater improvements in all primary outcomes (VAS, DHI, and SSS) compared with the conventional care group, with large effect sizes for the interaction effects (partial η^2^ range: 0.348–0.413).

### 3.3 Secondary outcome measures

In the secondary outcome analysis, we assessed VAS, DHI-T, and SSS scores at 1 month to evaluate the short-term effects of the intervention. As shown in Tables 3, 4, at 1 month, the combined intervention group demonstrated significantly greater improvements in the primary outcome measures (VAS, DHI, and SSS) compared with the conventional care group (*p* < 0.05). Between-group comparisons of the DHI subdimensions further revealed that the combined intervention group showed significantly greater improvements in the Emotional (DHI-E, *p* = 0.012) and Functional (DHI-F, *p* = 0.004) subdimensions, whereas the improvement in the Physical subdimension (DHI-P) did not differ significantly between groups, with no statistically significant difference (*P* > 0.05). At 3 months, the between-groups mean differences for the DHI subscales were 3.82 (95% CI: 2.762–4.878) for DHI-P, 6.78 (95% CI: 5.099–8.461) for DHI-E, and 5.34 (95% CI: 3.795–6.885) for DHI-F.

**Table 3:** VAS and SSS scores at baseline, 1 month, and 3 months by study group.

Scale	Group	Number of examples (*n*)	Baseline	1 month	3 months
VAS	Conventional care group	*n* = 50	4.02 (1.04)	2.98 (1.13)^*^	2.52 (1.02)^*#^
	Combined intervention group	*n* = 50	3.70 (1.07)	2.02 (1.06)^*^	0.14 (0.35)^*#^
	F		2.292	19.141	245.624
	P		0.133	<0.001	<0.001
SSS	Conventional care group	*n* = 50	44.22 (6.58)	40.44 (6.25)^*^	37.18 (6.41)^*#^
	Combined intervention group	*n* = 50	42.36 (7.45)	34.06 (6.67)^*^	24.48 (5.10)^*#^
	F		1.751	24.376	120.188
	P		0.189	<0.001	<0.001

Data are presented as mean (SD). VAS, Visual Analogue Scale; SSS, Somatic Symptom Scale.

1. Two-way repeated-measures ANOVA (time-by-group interaction and main effects).

VAS: Interaction *F*(1.968, 192.826) = 68.854, partial η^2^ = 0.413; Time main effect *F*(1.968, 192.826) = 397.228, *p* < 0.001, partial η^2^ = 0.802; Group main effect *F*(1, 98) = 53.408, *p* < 0.001, partial η^2^ = 0.353.

SSS: Interaction *F*(1.772, 173.629) = 52.367, *p* < 0.001, partial η^2^ = 0.348; Time main effect *F*(1.772, 173.629) = 274.319, *p* < 0.001, partial η^2^ = 0.737; Group main effect *F*(1, 98) = 37.913, *p* < 0.001, partial η^2^ = 0.279.

2. Within-group pairwise comparisons.

*p < 0.05 vs. baseline within the same group; #*p* < 0.05 vs. 1-month follow-up within the same group.

3. Between-group mean differences (Conventional care minus combined intervention) and 95% CI at each time point.

VAS: 1 month = 2.16 (1.668, 2.652), 3 months = 2.38(2.079, 2.681).

SSS: 1 month = 6.38 (3.816, 8.944), 3 months = 11.865 (9.873, 13.857).

4. ANCOVA results adjusted for age, educational level, disease duration at first consultation (in months), and baseline SSS score. Adjusted mean of conventional care group = 36.762 (95% CI: 35.363–38.162); adjusted mean of combined intervention group = 24.898 (95% CI: 23.497–26.297); adjusted mean difference = 11.865 (95% CI: 9.873–13.857), *F*(1,94) = 139.852, *p* < 0.001, partial η^2^ = 0.598.

**Table 4:** DHI total score and subscale scores at baseline, 1 month, and 3 months by study group.

Scale	Group	Number of examples (*n*)	Baseline	1 month	3 months
DHI-T	Conventional care group	*n* = 50	52.24 (12.90)	41.50 (11.20)^*^	29.70 (8.73)^*#^
	Combined intervention group	*n* = 50	51.82 (12.69)	34.96 (9.63)^*^	13.76 (7.70)^*#^
	F		0.027	9.808	93.700
	P		0.870	0.002	<0.001
DHI-P	Conventional care group	*n* = 50	13.56 (4.89)	10.58 (4.11)^*^	8.16 (2.80)^*#^
	Combined intervention group	*n* = 50	13.74 (3.42)	9.64 (3.32)^*^	4.34 (2.52)^*#^
	F		0.046	1.583	51.371
	P		0.831	0.211	<0.001
DHI-E	Conventional care group	*n* = 50	16.48 (5.71)	13.92 (4.91)^*^	9.98 (4.31)^*#^
	Combined intervention group	*n* = 50	16.60 (6.02)	11.56 (4.32)^*^	3.20 (4.16)^*#^
	F		0.010	6.498	64.027
	P		0.919	0.012	<0.001
DHI-F	Conventional care group	*n* = 50	22.20 (6.65)	17.00 (5.74)^*^	11.56 (4.49)^*#^
	Combined intervention group	*n* = 50	21.48 (7.70)	13.76 (5.07)^*^	6.22 (3.18)^*#^
	F		0.251	8.945	47.049
	P		0.618	0.004	<0.001

Data are presented as mean (SD). DHI-T, Dizziness Handicap Inventory-Total; DHI-P, DHI-Physical subscale; DHI-E, DHI-Emotional subscale; DHI-F, DHI-Functional subscale.

1. Two-way repeated-measures ANOVA (time-by-group interaction and main effects).

DHI-Total: Interaction *F*(1.414, 138.549) = 67.270, *p* < 0.001, partial η^2^ = 0.407; Time main effect *F*(1.414, 138.549) = 1013.236, *p* < 0.001, partial η^2^ = 0.912; Group main effect *F*(1, 98) = 14.805, *p* < 0.001, partial η^2^ = 0.131.

DHI-P: Interaction *F*(1.656, 162.286) = 29.919, *p* < 0.001, partial η^2^ = 0.234; Time main effect *F*(1.656, 162.286) = 384.996, *p* < 0.001, partial η^2^ = 0.797; Group main effect *F*(1, 98) = 5.511, *p* = 0.021, partial η^2^ = 0.053.

DHI-E: Interaction *F*(1.368, 134.016) = 43.407, *p* < 0.001, partial η^2^ = 0.307; Time main effect *F*(1.368, 134.016) = 358.320, *p* < 0.001, partial η^2^ = 0.785; Group main effect *F*(1, 98) = 11.346, *p* = 0.001, partial η^2^ = 0.104.

DHI-F: Interaction *F*(1.452, 142.281) = 14.982, *p* < 0.001, partial η^2^ = 0.133; Time main effect *F*(1.452, 142.281) = 469.571, *p* < 0.001, partial η^2^ = 0.827; Group main effect *F*(1, 98) = 9.201, *p* = 0.003, partial η^2^ = 0.086.

2. Within-group pairwise comparisons.

*p < 0.05 vs. baseline within the same group; #*p* < 0.05 vs. 1-month follow-up within the same group.

3. Between-group mean differences (Conventional care minus combined intervention) and 95% CI at each time point.

DHI-T: 1 month = 6.54 (2.396, 10.684), 3 months = 15.747 (13.513, 17.981).

DHI-P: 1 month = 0.94 (−0.543, 2.423), 3 months = 3.82 (2.762, 4.878).

DHI-E: 1 month = 2.36 (0.523, 4.197), 3 months = 6.78 (5.099, 8.461).

DHI-F: 1 month = 3.24 (1.090, 5.390), 3 months = 5.34 (3.795, 6.885).

4. ANCOVA results (adjusted for age, education, disease duration and baseline DHI).

Adjusted mean of conventional care group = 29.603 (95% CI: 28.028–31.179); adjusted mean of intervention group = 13.857 (95% CI: 12.281–15.432); adjusted mean difference = 15.747 (95% CI: 13.513–17.981), *F*(1,94) = 195.867, *p* < 0.001, partial η^2^ = 0.676.

Repeated measures analysis of variance revealed a significant time × group interaction effect for GAD-7 scores [*F*(1.590, 155.829) = 119.439, *p* < 0.001, partial η^2^ = 0.549] and PHQ-9 scores [*F*(1.456, 142.674) = 96.928, *p* < 0.001, partial η^2^ = 0.497], indicating that the combined intervention group and the conventional care group exhibited markedly divergent trajectories over time. At 3 months, the between-groups mean differences were 9.80 (95% CI: 8.712–10.888) for GAD-7 and 8.50 (95% CI: 7.152–9.848) for PHQ-9. The combined intervention group demonstrated a significant and sustained decrease in scores from baseline to 1 month and 3 months (*P* < 0.05), whereas the conventional care group exhibited a statistically significant deterioration over the same period. Therefore, at the 1 month and 3 months follow-up points after the intervention, the combined intervention group scored significantly lower than the conventional care group (*P* < 0.05). This finding suggests suggesting that the combined intervention was associated with alleviation of psychological distress and may have helped curb the natural progression of anxiety and depressive symptoms observed in the conventional care group, as shown in Table 5.

**Table 5:** GAD-7 and PHQ-9 scores at baseline, 1 month, and 3 months by study group.

Scale	Group	Number of examples (*n*)	Baseline	1 month	3 months
GAD-7	Conventional care group	50	6.02 (4.24)	7.74 (4.38)^*^	10.40 (3.65)^*#^
	Combined intervention group	50	6.82 (4.44)	3.26 (2.88)^*^	0.60 (1.31)^*#^
	F		0.849	36.553	319.698
	P		0.359	<0.001	<0.001
PHQ-9	Conventional care group	50	7.06 (4.61)	8.12 (4.58)	9.44 (4.44)^*#^
	Combined intervention group	50	8.28 (5.57)	4.26 (3.34)^*^	0.94 (1.85)^*#^
	F		1.425	23.208	156.49
	P		0.235	<0.001	<0.001

Data are presented as mean (SD). GAD-7, generalized anxiety disorder-7; PHQ-9, patient health questionnaire-9.

1. Two-way repeated-measures ANOVA (time-by-group interaction and main effects).

GAD-7: Interaction *F*(1.590, 155.829) = 119.439, *p* < 0.001, partial η^2^ = 0.549; Time main effect *F*(1.590, 155.829) = 4.799, *p* = 0.015, partial η^2^ = 0.047; Group main effect *F*(1, 98) = 53.472, *p* < 0.001, partial η^2^ = 0.353.

PHQ-9: Interaction *F*(1.456, 142.674) = 96.928, *p* < 0.001, partial η^2^ = 0.497; Time main effect *F*(1.456, 142.674) = 25.537, *p* < 0.001, partial η^2^ = 0.207; Group main effect *F*(1, 98) = 24.913, *p* < 0.001, partial η^2^ = 0.203.

2. Within-group pairwise comparisons.

*p < 0.05 vs. baseline within the same group; #*p* < 0.05 vs. 1-month follow-up within the same group.

3. Between-group mean differences (Conventional care minus combined intervention) and 95% CI at each time point.

GAD-7: 1 month = 4.48 (3.010, 5.950), 3 months = 9.80 (8.712, 10.888).

PHQ-9: 1 month = 3.86 (2.270, 5.450), 3 months = 8.50 (7.152, 9.848).

## 4 Discussion

This real-world cohort study systematically evaluated the CB-VR model—an integrated intervention combining cognitive behavioral therapy with vestibular rehabilitation training—for patients with vestibular dysfunction and negative emotions in routine clinical practice. Our primary finding is that the combined intervention group showed significantly greater improvements than the conventional care group across all outcomes, including dizziness severity (VAS), disability (DHI), somatic symptoms (SSS), and psychological distress (GAD-7, PHQ-9). Notably, our data revealed a temporal pattern in which most outcomes showed significant between-group differences at 1 month, whereas the DHI physical subscale reached significance only at 3 months. This temporal trajectory suggests that early CBT-induced emotional relief may lower patients’ fear of dizziness and movement avoidance, thereby enhancing their engagement with and responsiveness to subsequent vestibular rehabilitation training. Our findings align with expert consensus recommendations (33) for integrating psychological and rehabilitative care in this population, and extend them by providing real-world evidence that this model may be feasible and beneficial when delivered by nursing staff within routine clinical workflows—addressing the gap between controlled trial findings and clinical practice. The overall treatment effect observed in this study should be interpreted with caution, given the clinical heterogeneity of the included population (PPPD, Ménière‘s disease, and vestibular migraine). While the pooled analysis across diagnostic groups showed a consistent pattern of improvement, these conditions differ in their underlying pathophysiology and may respond differentially to CBT and vestibular rehabilitation. The observed effect should therefore be considered an average estimate across this heterogenous cohort, and may not be uniformly applicable to each individual subtype.

In the comparison of dizziness improvement, the combined intervention group demonstrated significantly greater improvement in VAS and DHI scores at 1 and 3 months compared to the conventional care group. Further analysis of DHI subscales revealed a clear temporal sequence: the CB-VR group’s advantages at 1 month mainly stemmed from improvements in the emotional (DHI-E) and functional (DHI-F) dimensions, whereas the significant advantage in the physical dimension (DHI-P) was not established until 3 months (*p* < 0.01). This temporal dissociation suggests that the combined intervention may first alleviate psychological distress and functional limitations, which could subsequently facilitate physiological recovery—consistent with our observation that between-group differences in most outcomes were observed as early as 1 month, whereas the DHI physical subscale showed a delayed separation at 3 months. While another study reported significant DHI total score improvements within 2–4 weeks of nurse-led peripheral vestibular rehabilitation (41), the slower physical recovery observed in our study is likely attributable to the inclusion of patients with central vestibular dysfunction—defined in this study as vestibular migraine and PPPD, both of which involve central vestibular processing pathways, in contrast to peripheral vestibular dysfunction (e.g., Ménière’s disease), which involves the peripheral end organs or vestibular nerve. Patients with central vestibular dysfunction generally require longer rehabilitation periods due to more complex neural compensation mechanisms. This difference highlights the need for distinguishing between peripheral and central etiologies when interpreting rehabilitation outcomes. More importantly, our findings suggest a potential therapeutic advantage of the CB-VR model: rather than accelerating physiological recovery per se, its primary value may lie in achieving early and sustained improvements in emotional well-being and daily functioning. One possible explanation is that early psychological relief may operate through two interrelated pathways—reducing illness-related catastrophizing and fear of movement, and restoring self-efficacy and motivation, thereby improving adherence to rehabilitation exercises over subsequent weeks. However, this mechanistic interpretation remains speculative and requires direct testing in future studies. From a clinical nursing perspective, this temporal pattern may offer some practical considerations for care sequencing: the first month should prioritize psychological support and functional goal-setting, with nurses actively addressing maladaptive cognitions about dizziness; as patients’ psychological state stabilizes, rehabilitation training can be progressively intensified during months 2–3 to capitalize on their improved readiness for physical exercise. This phased approach can be implemented within existing nursing workflows without additional specialist resources.

To assess improvements in physical symptoms, we employed two complementary scales—DHI and SSS—for further analysis. Patients in the combined intervention group showed significant improvement on the SSS scale as early as 1 month, whereas the advantage in the DHI-P only became apparent at 3 months. This temporal discrepancy likely reflects that the two scales capture different aspects of physical symptoms: the SSS focuses on acute physical discomfort such as nausea and headache, which are closely linked to autonomic nervous system responses, whereas the DHI-P assesses chronic, balance-related perceptions that may require a longer adaptation process. The early improvement in SSS suggests that the combined intervention may exert rapid benefits at the physiological–emotional interface, although the underlying mechanisms remain speculative and require further investigation. These findings suggest that a nurse-led CB-VR intervention may be associated with significant improvements in somatic symptoms, and that this improvement follows a time-dependent pattern, with early SSS gains followed by later DHI-P improvements. For clinical nursing practice, this temporal pattern suggests a phased assessment strategy: the SSS scale may be useful for monitoring early, emotion-related physical responses, while the DHI-P may be more appropriate for evaluating longer-term physical functional recovery. Analysis of negative emotions in both patient groups revealed that, due to cognitive and behavioral modifications, patients in the combined intervention group showed marked improvement in anxiety and depression scores compared with the conventional care group. However, patients receiving conventional care group exhibited a progressive deterioration in Generalized Anxiety Disorder Scale-7 (GAD-7) and Patient Health Questionnaire-9 (PHQ-9) scores. This suggests that long-term medication and health education alone may be insufficient to halt the psychological deterioration linked to the progression of chronic disease, and the difference between the two groups likely reflects the absence of targeted psychological interventions in routine care. In contrast, the combined intervention group strengthened patients’ self-efficacy and coping strategies through cognitive behavioral modification, establishing a positive psychological adaptation pattern. Consistent with the findings of Dunlap et al. (3, 42), our results further suggest that CB-VR may be associated with greater improvements in anxiety and depression compared to medication alone. By targeting both cognitive and physical aspects, this combined approach may help improve the interrelationship between irrational cognitions, negative emotions, and balance instability, potentially offering psychological benefits beyond those of conventional care. For nursing practice, these findings suggest that nurses may consider incorporating psychological assessment into early screening protocols and initiating cognitive-behavioral interventions for patients with elevated GAD-7 or PHQ-9 scores, rather than waiting for medication to take effect, supporting the integration of psychological care into routine vestibular rehabilitation nursing. Whether this model is scalable or cost-effective as a workforce solution requires formal implementation and health economic evaluations, which were not examined in this study. Future research is needed to assess these aspects before any claims regarding workforce scalability can be made. Collectively, these findings suggest that the nurse-led CB-VR model offers added value beyond medication and health education, and suggest that its mechanism of action may differ from that of conventional rehabilitation training alone, potentially through its focus on the psychological and functional dimensions that are often overlooked in standard care. The large effect sizes observed in this study warrant careful interpretation. The combined intervention involved substantially more structured contact than usual care (3 structured sessions, 60–90 min each, total 3–4.5 h), which may have contributed to greater expectancy effects and attention—related benefits. All outcomes were based on self-report questionnaires, which may be influenced by social desirability bias. Although baseline scores were balanced between groups, the control group showed worsening over time, suggesting that regression to the mean is unlikely to be the primary explanation for the observed between-group differences. Adherence was high in the intervention group, whereas adherence in the control group was not formally measured; this difference may have contributed to the observed effect sizes. These factors should be considered when interpreting the magnitude of the reported improvements.

This study has several limitations. First, the single-center, retrospective cohort design may be subject to selection bias and confounding by indication, as treatment allocation was based on standardized baseline assessments and whether patients actually initiated the intervention, rather than randomization. In addition, although the attrition rate was low (2.9%), the possibility of attrition bias cannot be entirely excluded. The sample size within each diagnostic subgroup was too small to perform meaningful stratified analyses, and the clinical heterogeneity of the cohort (PPPD, Ménière’s disease, and vestibular migraine) limits the interpretability of the overall effect, as these conditions may respond differently to the intervention. Although the diagnostic distribution was balanced between the two groups at baseline, future studies with larger samples are needed to explore whether treatment effects differ across specific vestibular diagnoses. Second, the 3-month follow-up period precludes assessment of the long-term sustainability of the intervention effects. Third, the combined intervention involved more structured contact than conventional care, making it difficult to separate the specific effect of CBT from nonspecific attention effects, and the specific contributions of CBT, vestibular rehabilitation, or their combination cannot be isolated from the current study design, and treatment fidelity was not formally assessed. Furthermore, the study was not prospectively registered, and the evaluation of multiple outcomes across multiple time points introduced a multiplicity burden. No adjustment for multiple testing across outcomes or subscales was performed, and findings related to secondary outcomes and subscales should therefore be considered exploratory and hypothesis generating. Therefore, our findings should be interpreted as exploratory real-world evidence. Nonetheless, this study provides preliminary real-world evidence supporting the feasibility of the nurse-led CB-VR model in improving psychological symptoms and functional outcomes in patients with vestibular dysfunction and negative emotions. Future studies may consider active control designs (e.g., general supportive sessions or attention-matched interventions) to disentangle the specific effects of CBT from non-specific attention effects, along with larger samples, longer follow-up, and pre-registration to confirm and extend our findings. Finally, as treatment was not randomly assigned, residual confounding cannot be ruled out. Important factors such as patient motivation, access to treatment, clinician referral, adherence, and socioeconomic circumstances were not measured and may have influenced the observed associations. Therefore, our findings should be interpreted as associative rather than causal.

## 5 Conclusion

In summary, within the context of real-world clinical care, cognitive behavioral therapy combined with personalized rehabilitation training was associated with superior improvements in psychological outcomes for patients with vestibular dysfunction and negative emotions compared with conventional care (medication and health education). This approach was associated with reductions in the severity of subjectively perceived dizziness and body-related symptoms, as well as improvements in functional disability. These findings suggest that the integrated care model warrants further prospective evaluation in larger, adequately powered studies to assess its feasibility, generalizability, and cost-effectiveness. However, as the specific contributions of CBT, vestibular rehabilitation, or their combination cannot be determined from this study design, these findings should be interpreted with caution and confirmed in future studies using dismantling designs or active comparators.

## Data Availability

The raw data supporting the conclusions of this article will be made available by the authors, without undue reservation.
